# Comparing machine learning models with a focus on tone in grooming chat logs

**DOI:** 10.3389/fped.2025.1591828

**Published:** 2025-06-19

**Authors:** Leonie Hamm, Steve McKeever

**Affiliations:** Department of Informatics and Media, Uppsala University, Uppsala, Sweden

**Keywords:** grooming detection, online predators, sentiment analysis, large language models, predator tone, social exchange theory

## Abstract

In online spaces, children are vulnerable to exploitation and sexual predators. Groomers contact minors in online chat rooms with the intent of sexual abuse. This study investigates how new deep learning models compare to traditional machine learning models in detecting grooming conversations and predatory authors. Furthermore, we detect the underlying tones used by predators and explore how these affect detection capabilities. Our goal is to better understand predator tactics and to advance automatic grooming detection in order to protect children in online spaces. The PAN12 chat logs, which contain grooming chat conversations, were used as the dataset for the research. These chat conversations were sorted into sentiments through the DistilBERT classifier based on the predator tone. SVMs and the LLaMA 3.2 1B large language model by Meta were then trained and fine-tuned on the different sentiments. The results measured through precision, recall and $F_{1}$ score show that the large language model performs better in grooming detection than traditional machine learning. Moreover, performance differences between the positive and negative sentiment are captured and indicate that positive tone improves detection while negative toned grooming conversations have nuanced patterns that are harder to distinguish from non-grooming. This shows that groomers employ varying strategies to gain access to their victims. Lastly, with an $F_{1}$ score of 0.99 and an $F_{0.5}$ score of 0.99, the LLaMA 3.2 1B model outperforms both traditional machine learning, as well as previous versions of the large language model in grooming author detection.

## 1 Introduction

The Internet has become an indispensable part of our lives, giving us access to information, entertainment, and communication. However, it poses risks and dangers, particularly to children. Although many social media platforms require their users to be at least 13 years old, they do not ask for a date of birth during registration, and children and teens can freely register without problems or supervision. Surveys show that in 2023, 96% of US teens (aged 13–17) used social media platforms daily (1). By spending time in online spaces, teens and children often come into contact with strangers, sometimes sex offenders. Text messages through social media platforms can often be the first step for predators to contact their victims. Social media and chat rooms are ways for predators to reach children, communicate with them, initiate inappropriate conversations, and are used by predators to initiate sexual relations with minors.

This action of getting in contact with minors with the intent of sexual abuse is called grooming (2, 3). During the grooming process, the predator tries to establish an emotional relationship with the targeted child, and recent research shows that “nearly half of the offenders who had committed one or more contact offenses, i.e., they had directly or physically abused children, had displayed so-called *grooming behavior*” [(4), p. 3–4]. Through exploitation, inappropriate relationships, and the spread of child sexual abuse material (CSAM), children are victimized and harmed, usually leading to emotional trauma that can cause long-lasting psychological problems (5).

With the growth of social media, new solutions must be found to protect children from predators on the internet. The large amount of data no longer allows manual detection of grooming and exploitive conversations. The automatic detection of inappropriate conversations between adults and minors becomes essential to children’s online safety. The research that has been conducted on the automatic detection of child exploitation material is mostly focused on child sexual abuse material such as pornography. Focusing on detecting grooming conversations helps protect children from abusive situations, that CSAM emerges from. Moreover, chats that are detected as grooming conversations can be used to convict predators.

Since online chat rooms are a means for offenders to initiate contact with children, the analysis of those initial conversations helps to detect predatory and grooming behavior. While accurately detected predatory chats can be used as evidence to prosecute a predator, existing research does not attempt to understand the underlying structures of the grooming conversations. Our work attempts to discover some of these underlying patterns by examining the tone of voice used by predators. Moreover, little research has been undertaken to try and understand how the developed detection models capture grooming conversations and in which cases they struggle. By addressing this research gap, machine learning models can help to understand structures and strategies used by groomers. A better understanding of how predators act essentially leads to the possibility of developing better grooming detection tools to protect children.

This paper aims to examine machine learning algorithms and deep learning models and their ability to detect grooming patterns in chat logs. Models based on recent developments are compared with the algorithms used in existing research. Furthermore, the tone of voice as a behavioral pattern and its importance in the detection of grooming conversations is examined. In this paper, the following research questions will be examined:

• How does deep learning compare to traditional machine learning in detecting grooming conversations in chat logs?
• Does the tone used by predators impact the effectiveness of grooming conversation detection in chat logs?
• How do newly released large language models compare to those developed by other researchers in detecting grooming?

To compare deep learning with traditional machine learning, one representative, state-of-the-art model of each category is selected to exemplify deep learning and traditional machine learning. For detecting grooming conversations, this comparison is valuable because each approach offers distinct advantages and limitations. Moreover, the stakes in this application are extremely high, requiring a thoughtful selection of methods. Traditional ML performs well with structured data and small to medium-sized datasets. Whereas deep learning automatically learns complex patterns and context from raw text, such as nuanced emotional tone, manipulation tactics, or disguised intent. It is particularly powerful with large datasets and unstructured data. However, the generated artifacts are more opaque. Deep learning requires more computational resources, training time, and data. Traditional ML is often easier to deploy and maintain in low-resource environments. Comparing the two helps decide whether to use one over the other, or to combine them. A rigorous comparison ensures that the chosen model balances accuracy, fairness, and feasibility—all crucial in grooming detection where false positives can damage reputations and false negatives can leave children at risk.

To analyze the tone predators use, the chat logs are separated into positive and negative toned conversations through sentiment analysis. To further analyze how tones impact the effectiveness of grooming conversations, models are trained on specific tones to detect predatory conversations in chat logs. For the training of these models, the same kinds of models are used as when comparing deep learning with traditional machine learning. Since the models resulting from answering our first research question are not tone-specific, they serve as a baseline for evaluating the effectiveness of subsequent tone-specific models. Insights into how tone is used by predators and how grooming detection classifiers react to those tones help us understand predators’ behaviors more, bringing us one step further in protecting children online.

Lastly, to answer the third research question, a newly released large language model is implemented and the performance results are compared to the performance of other researchers’ models. Here, the task is to detect predatory authors, as opposed to the task of detecting predatory conversations. By comparing a newly released model with the models that were implemented by other researchers in the past, we aim to advance the methods that are used to identify harmful behavior in text messages.

To answer all research questions, the effectiveness and the ability of a model to detect grooming are measured through multiple established performance metrics. These metrics include precision, recall and $F_{\beta }$ scores. This paper is structured as follows. In Section 2 we discuss previous work in grooming detection and the various machine learning models used. In Section 3 we introduce the social science theory that will be used to explain our results, how to evaluate approaches using standard metrics, and present the various phases of our analysis pipeline. In Section 4 we present the results of our experiments, including the effect of tone, and discuss their implications in Section 5. Finally, in Section 6 we summarize our study.

## 2 Background

In this section we summarize the literature with regards to the topic of grooming detection in chat logs. Different strategies of grooming detection are presented, along with commonly used datasets and machine learning algorithms.

Grooming is a problem that is not uncommon in online environments. However, to understand grooming, its progression, and how to detect it, the term needs to be clearly defined. A widely adopted definition of grooming is given by Gillespie (6) that describes grooming as:

“the process by which a child is befriended by a would-be abuser in an attempt to gain a child’s confidence and trust, enabling them to get the child to acquiesce to abusive activity” [(6), p. 411].

Unlike other harmful online behavior, such as cyberbullying and harassment, grooming is based on manipulative techniques that trick children into relationships with the predator (7). Important elements of this manipulation are gaining access to the victim, ensuring they do not talk to parents or other adults about the relationship, and gaining obedience (8). Predators often conduct the grooming process in stages that build trust and isolate the child from their environment to then introduce sexual topics to the conversation.

In most existing research papers, developing grooming detection models is performed on labeled datasets, where the conversations, chats, or authors have corresponding labels that indicate whether they show predatory behavior or not. In grooming detection datasets, the labels are typically “predatory” or “non-predatory” and are given manually by experts. When a dataset with labels is used for grooming detection, the classification task is seen as a supervised learning problem. In supervised learning, the model learns from labeled instances and then predicts the label for new, unknown instances (9). Ebrahimi et al. (10) implemented a semi-supervised model for grooming detection by training an anomaly detector only on the positive, predatory, class.

In Borj et al. (11), the authors note that there are three main approaches when it comes to grooming detection research. The first approach is to detect the different stages that characterize the grooming process in online chat logs. By discovering these stages in text messages, grooming can be caught in the early stages of a conversation. The goal of this approach is to detect grooming behavior as soon as possible. This approach is not further pursued in this research as it is harder to incorporate predator tone in the analysis. Predator tone emerges over the course of the conversation and is not always immediately obvious. A second approach is to detect predatory conversations and predatory authors in chat logs as accurately as possible, regardless of how far the conversation has progressed. Here, the goal is to detect as many groomers as possible while ensuring that the detected groomers are correctly identified and show, in fact, predatory behavior. This approach is used in this research as it provides the possibility to analyze whole conversations and the tone used in them. A final approach is not based on their interactions and behavior in chat conversations, but instead on their personal profiles. Aspects such as demographic attributes (e.g., age and gender), personality, and behavioral characteristics are extracted from text written by the author and analyzed. As our research focuses on grooming detection through interactions in chat logs and the tone that groomers employ in these conversations, author profiling is not a focus of this thesis.

Online grooming commonly occurs in private chatrooms Borj et al. (11). To implement grooming detection models, such private chat logs need to be inspected. Online chat conversations containing grooming behavior are rare and cannot be easily accessed as one-on-one online chats are not visible to the public. Moreover, conversations between predators and children are not accessible to protect children from further emotional trauma and exploitation. Perverted Justice (PJ) is a foundation that operated until 2,019 with the purpose of convicting online predators based on chat conversations (see http://www.perverted-justice.com/). Police officers that worked for PJ pretended to be children, in order to lure predators to then prosecute them. During Perverted Justice’s years in operation, they achieved over 600 convictions. PJ made the chat logs between predators and police officers openly accessible to the public (see https://zenodo.org/records/3713280). These chat logs are the base data used in the majority of grooming detection research (12–14). They serve as the positive predatory class and have been used by Cook et al. (15) to demonstrate that machine learning algorithms are less effective than humans at identifying 11 known communication strategies. They argue that accurate detection of these strategies requires human annotators to validate and interpret the outputs of the machine learning models. Another source for predatory conversations, which is not publicly available, is MovieStarPlanet as used in research by Cheong et al. (16). MovieStarPlanet is a platform for online games that also allows users to chat with each other. MovieStarPlanet therefore provides a tool for predators to specifically target children. Another approach that is not based on publicly available data is that by Ngo et al. (17). They were able to acquire predatory chat logs through collaboration with their national reporting center for child sexual abuse and Web-IQ, a company that combats online child abuse. The dataset stems from the dark web and is labeled manually by experts.

Preprocessing the datasets (12) can increase the performance of grooming detection models significantly. Such techniques implemented by researchers include the removal of stop-words, stemming, and tokenization, to remove noise from the dataset (12). By removing stop-words, words that do not carry a significant meaning, the focus is put onto words that are more relevant for the context and understanding. Stemming reduces words to their root so that words with the same meaning are treated as one. Tokenization breaks the text into smaller units. Conversations that have only one author or more than two authors can be filtered out as they rely on the assumption that the predator will contact the victim in a private one-on-one chat. Similarly, only conversations in the dataset where each author sent at least seven messages are kept. Borj et al. (12) argue that shorter conversations lack sufficient information to deduce whether it is a predatory or non-predatory conversation.

The PAN12 dataset was created for the Sexual Predator Identification competition organized in 2012 by PAN (18). The goal of the competition was to correctly detect groomers in online chat conversations. The PAN12 chat logs are structured as conversations, where each conversation has one or multiple messages and each message contains an anonymized author tag, a timestamp, and text. The predatory chat logs in the PAN12 dataset are provided by the Perverted Justice Foundation. These chat logs are the result of police officers pretending to be minors to get into contact with predators and to entrap them. Furthermore, it includes non-predatory chats, as well as chat conversations that are taken from Omegle. Omegle was a website used for connecting adults and chat logs included from Omegle are of sexual character to cover the challenge of trying to differentiate sexual conversations between adults from grooming conversations with minors (2). Some of these Omegle conversations are labeled as non-predatory but may still contain grooming behavior, as labeling was done based on source rather than content. Due to the small amount of predatory chats compared to the large amount of non-predatory chats, the PAN12 has the issue that it is unbalanced. Merely 3% of conversations have a groomer present. However, imbalance can be overcome through proper preprocessing and feature extraction techniques. While the PAN12 dataset is over ten years old, it provides a good baseline to compare different machine learning models. Having said that, an older dataset might not accurately reflect current grooming language or tactics. Online communication evolves rapidly—groomers may adapt their strategies to avoid detection or to exploit emerging social norms, slang, and technologies.

### 2.1 Feature extraction and classifier approaches

Feature extraction is the encoding of information into numerical vectors that can be processed by machine learning models. In the context of grooming detection, the information present in the chat logs is represented by these feature vectors (11). Different options for feature extraction are Bag of Words models (BoW) like Term Frequency (TF) or Term Frequency—Inverse Document Frequency (TF-IDF) which are based on word frequencies or Word Embedding models (e.g., Word2Vec) which try to capture context by mapping words into a vector space (19). Another option for feature extraction is through the Simple Contrastive Sentence Embedding framework (SimCSE) as implemented by Borj et al. (20). In this framework, feature extraction is undertaken based on pre-trained language models such as the BERT (Bidirectional Encoder Representations from Transformers) series to improve the quality of the embeddings (21). Both Milon-Flores and Cordeiro (22) and Fauzi et al. (13) successfully investigate whether the extraction of behavioral and emotion-based characteristics can improve the classification performance of their models.

Various machine learning algorithms have been used to implement classifiers that detect predatory chats and predatory authors. The most commonly used traditional machine learning algorithms are Support Vector Machines (SVM), Naïve Bayes (NB), k-Nearest Neighbors (KNN), Random Forest (RF), logistic regression, and decision trees (12, 13, 16, 23, 24). Furthermore, Ebrahimi et al. (25) have utilized deep learning techniques in the form of Convolutional Neural Networks (CNN).

### 2.2 Natural language processing

Natural language processing (NLP) focuses on developing artificial intelligence that can understand, interpret, and analyze human-like text (26). Over the last few years, large language models have emerged and gained popularity. They show outstanding performance in various natural language processing (NLP) tasks, such as classification tasks or text generation (26). This is because they understand context in human-like text and are able to solve more complicated tasks than traditional machine learning models. Examples of well-known LLMs are the GPT series by OpenAI, BERT by Google, LLaMA by Meta, or ChatGLM (26). Most state-of-the-art large language models (LLMs) are built on the transformer architecture (27), which leverages a self-attention mechanism to capture dependencies over larger text sequences. This mechanism enables the model to assess the relative importance of each element in a sequence in the context of all other elements. Moreover, because they process tokens in parallel, transformer-based LLMs can be scaled efficiently (28). These structures lead to the dominance of LLMs in NLP tasks. However, transformer-based LLMs require high computational power and use lots of memory because of the complexity of their attention mechanism.

The majority of new large language models are pre-trained on a massive corpus of unlabeled text to gain a general understanding of the grammar, semantics, and patterns of language. After pre-taining, the models can then be fine-tuned to align themselves to a specific task (29). This allows for a faster training process while maintaining the performance of a model trained on vast amounts of data that already has an overall understanding of language. More specific patterns of the language are captured by fine-tuning the model on a smaller amount of data that is related to the NLP task.

An attempt by Nguyen et al. (14) to detect grooming in chat logs through an LLM was undertaken. They fine-tuned the model on the PAN12 dataset for the task of grooming chat detection. Two prefiltering steps were implemented: only conversations with two authors were kept and conversations with less than seven messages per author were removed. These steps align with previous research where grooming detection is undertaken with traditional machine learning models. Non-English words, however are kept, as the LLaMA 2 model can handle noisier text better than traditional models.

### 2.3 Challenges in existing research

Predators that contact their victims through online chat logs have different goals. This leads to a wide variety of chat conversations and predatory authors. Machine learning models often struggle to generalize across such diverse conversations and accurately identify predatory behavior (15). Furthermore, chat logs from online platforms can be quite noisy. Abbreviations and slang are commonly used, and chat logs frequently include misspellings. Another problem that needs to be faced by researchers is the imbalance between predatory and non-predatory chats. Predatory conversations are rare compared to harmless chats. Finally, technology constantly improves through ongoing research and innovations, large-language models are evolving rapidly. Leading AI companies such as OpenAI, Meta, or Google release new versions of their LLMs frequently, roughly every 6 to 12 months, so published research in detecting predatory grooming behavior will often be based on obsolete models.

### 2.4 Model selection

In grooming detection, choosing the right models is a crucial step to achieve meaningful results. The chosen models should not only be accurate when detecting conversations as predatory, but should also help to gain an understanding of the patterns that predators use. The PAN12 consists of online chat logs and also provides labels that indicate whether an author is a groomer and whether a specific conversation shows predatory behavior. To deal with a labeled dataset, supervised learning is employed. Two primary NLP approaches are followed in this research, grooming detection through traditional machine learning and grooming detection through state-of-the-art deep learning. These two text classification models were chosen based on their compatibility with the PAN12 dataset and the objective of detecting grooming in chat logs. Previous surveys of different models’ performances on the PAN12 dataset provided additional insight in choosing the right models (11).

To understand the linguistic patterns and strategies of groomers, specifically when it comes to the tone of voice, a model has to be implemented that detects whether a conversation is written in a positive tone or negative tone. As the research in understanding tone patterns is built on these labels, the success of the analysis depends on the model that is selected for the sentiment analysis. In the following sections, the choices for the traditional machine learning model, the deep learning model, and the sentiment analysis model are explained further.

### 2.5 Traditional machine learning model

Traditional models that are used for NLP tasks include Naïve Bayes (NB), K-nearest neighbor (KNN), Support Vector Machines (SVM), and Decision Trees (DT) (30). The Naïve Bayes classifier works under the assumption that the input features are independent of each other. In grooming conversations, messages and words are often connected through context, which makes the features are non-independent. Therefore, Naïve Bayes is not ideal for the PAN12 dataset. While Naïve Bayes has shown good results in research on grooming detection, it is outperformed by alternative machine learning models (see Tables 1, 2). Moreover, PAN12 is a rather large dataset. To accomplish efficient training, a classifier needs to be implemented that does not require large computing resources and time. The KNN classifier does not train on a model in the traditional sense. Instead, the algorithm remembers the whole dataset and when presented with a new element that needs to be classified, the classifier compares it to the dataset. The prediction then is based on the distance to each data point (30). Because of the lack of a model and the need to store the training dataset, the KNN classifier would require a long time for computation and a lot of memory. It is therefore not suitable for grooming detection with the PAN12 dataset either.

**Table 1:** The best-performing approaches within grooming detection.

Reference	Detection task	Feature extraction	Classifier
Fauzi and Bours (24)	Predators	BoW (TF, Binary features)	Multinominal NB, Bernoulli NB
Borj et al. (23)	Predators	Word2Vec	Histogram gradient boosted decision tree
Borj et al. (20)	Grooming chats	SimCSE	Fusion (SVM, RF, NB, SGD)
Fauzi et al. (13)	Predators	BoW (TF-IDF)	SVM
Nguyen et al. (14)	Grooming chats	No feature extraction	LLaMA 2 7B

**Table 2:** Performance results of the best approaches.

Reference	Accuracy	Precision	Recall	$F_{1}$	$F_{0.5}$
Fauzi and Bours (24)	–	0.96	0.86	0.90	0.93
Borj et al. (23)	0.99	–	–	**0.99**	0.94
Borj et al. (20)	0.99	0.99	0.96	0.97	**0.98**
Fauzi et al. (13)	0.98	0.98	0.98	0.98	**0.98**
Nguyen et al. (14)	1.00	–	–	0.98	**0.98**

Bold values indicate the highest values in a particular column.

Decision Trees are intuitive models with a tree-like structure. To make predictions, features of a new input element are compared to a threshold at each node, and the final decision to determine the predicted class is made by following the path until the leaf node (30). Decision trees are suitable for unbalanced datasets as the weights of each path can be adjusted so that the minority class is assigned a higher weight (31, 32). However, decision trees have a similar problem as KNN and can become inefficient when dealing with a large training dataset (33). Moreover, they are prone to overfitting, which can become a huge problem when dealing with a highly imbalanced dataset such as PAN12 (34). Similarly, Random Forests, although effective and less prone to overfitting, can become computationally expensive compared to other approaches as they build upon the Decision Tree model.

Classification through SVMs is achieved by finding a hyperplane that best separates the elements of the different classes (30). SVMs are traditionally designed for binary classification and can be implemented with linear and non-linear kernels. While the usage of a linear kernel leads to faster execution and needs less computing power, a non-linear kernel is valuable to understanding more complex and heterogeneous patterns (35). Additionally, SVM work well together with feature extraction methods that transform the raw input data into numerical vectors, which capture the most important features of the data (13). The language that is used by predators in the PAN12 dataset is often complex as it can employ strategies like manipulation. Therefore, using an SVM with either a linear kernel for faster execution or a non-linear kernel for a better understanding of complex patterns, in combination with an appropriate feature extraction method is a suitable strategy for building a robust grooming detection model that can detect patterns used by predators. Groomers, but also non-groomers, can show a variety of distinct behaviors, which may challenge the assumption that all conversations can be separated into two homogeneous classes. However, experimentation with non-linear and linear kernels helps with addressing these complexities. Despite these limitations, SVM are commonly implemented by researchers when working with grooming detection on the PAN12 dataset (11).

### 2.6 Deep learning model

Deep learning is a subcategory of machine learning that is based on artificial neural networks that consist of many layers. Large language models are deep learning models that can be implemented to solve natural language tasks as they are trained to understand language (36).

The latest and most powerful LLM families include but are not limited to GPT by OpenAI, LLaMA by Meta, and Gemma by Google AI (36, 37). All of these model families are based on the transformer architecture, which implements a self-attention mechanism (36, 38). These self-attention layers calculate the relation and importance of each word to all other words in the sequence, enabling the model to capture contextual patterns and meaning (27). Because of their understanding of long-range relationships in sequences, these LLMs are typically used to solve natural language tasks where the language is complex and nuanced (39) making them relevant for tasks like grooming detection. GPT, LLaMA, and Gemma, are models that are pre-trained on trillions of unlabeled tokens from mainly online sources and documents to understand language patterns, semantics, and grammar for general NLP tasks (36, 40). From there the models can be fine-tuned to more specific tasks with smaller amounts of labeled data (39).

While the performance of an LLM generally improves through the increase in the number of parameters, the amount of time required to fine-tune the model also increases. For the scale of this research, the time that is needed for fine-tuning a model has to be in relation to the improvement in performance. The objective of this research is to compare the performance of traditional machine learning to new deep learning models. This goal can be reached without implementing the largest and best version currently available. Choosing to fine-tune an LLM with fewer parameters makes the two approaches more comparable as the time required for training or fine-tuning is more alike.

Meta released their newest LLM LLaMA 3.2 in September 2024 with a text-only lightweight version that has one billion parameters, drastically reducing the computational power and memory needed to fine-tune this model (41). LLaMA 3.2 1B is a recently released model, that has a manageable size while retaining a high performance. Because of these factors and because it is openly available for use, the LLaMA 3.2 1B model is the best choice as a deep learning model for comparison with traditional machine learning models in the context of grooming detection.

### 2.7 Sentiment analysis model

One objective of this research is to examine how groomers employ tone of voice and what impact that has on grooming detection performance. Therefore, it is necessary to perform a sentiment analysis. During this analysis, all conversations were sorted into groups based on what tone of voice was used. BERT, a self-supervised NLP model, was chosen for the sentiment analysis as it can understand complex language and patterns without needing a labeled dataset (42). Pre-trained BERT models are available that are trained on large corpora of data (BooksCorpus and English Wikipedia) and that can be implemented for various language processing tasks (36). Unlike traditional lexicon-based models, BERT understands context through its bidirectional mechanism. The mechanism enables the encoder to read a complete sequence at once during pre-training instead of from left to right (43). Due to its understanding of context and superior performance over traditional lexicon-based methods, it is now widely adopted for sentiment analysis (44).

Grooming conversations are often nuanced, they demonstate both complex language and manipulative behavior. BERT can capture these complexities in the language based on its ability to understand the context of a word, making it a good choice for sentiment analysis in the context of understanding the tone of voice in these online conversations. A disadvantage of BERT is that models are big and require a long time to run a sentiment analysis. DistilBERT is a smaller version of a BERT model with the same structure but with fewer parameters. The DistilBERT model keeps 97% of the original BERT model’s language understanding while being 60% faster (45). Due to limited available computing power, limited memory, and a large dataset, the DistilBERT model is the best option to perform sentiment analysis on the PAN12 dataset.

## 3 Methodology

In this section we present relevant theory used to motivate our study, explain how to evaluate machine learning models, discuss the efficacy of existing approaches, and present our research setup.

### 3.1 Social exchange theory and investment model

Social exchange theory (SET) is a sociological and psychological framework for studying interactions between two parties and was first introduced by Homans (46). It views relationships through a cost-benefit analysis and states that individuals enter a relationship or interaction based on their perception of risks and benefits. Therefore, individuals continuously calculate consciously but also unconsciously if it is worth entering, staying, or leaving a relationship. The social exchange theory explains that individuals try to maximize the benefits and minimize the cost in relationships. Relationships as seen through the social exchange theory can be romantic relationships, friendships, work relations, or simple interactions with strangers in everyday life. Individuals leave relationships when the perceived effort outweighs the benefits (47).

The two main concepts of the social exchange theory are costs and benefits. Costs are the aspects of the relationship that the individual perceives as negative. Examples of the costs are money, emotional strains, or other sacrifices. In contrast, the benefits are the rewards of the relationship. They are seen as positive outcomes of the relationship. Benefits can be affection, attention, emotional and social support, or materialistic aspects. Costs and benefits are perceived subjectively and differ from individual to individual. One’s judgment is influenced by expectations that are formed through past experiences and cultural norms. If someone has lower expectations they are more likely to enter or stay in relationships that others consider to have high costs and low benefits (46). Moreover, while weighing costs and benefits, the availability and quality of alternatives also play a role in the calculations on whether to enter or stay in a social relationship. The fewer qualitative alternatives exist, the higher the commitment (47).

Rusbult (48) developed the investment model based on the social exchange theory to address the shortcomings of SET. Similarly to SET, the investment model puts emphasis on commitment, which is described as the desire and intention to continue a relationship. The investment model consists of three main factors that determine the level of commitment: satisfaction, quality of alternatives, and investment. The satisfaction can be described as a comparison of rewards and costs as defined by Homans (46). Moreover, individuals evaluate if there are better options available to them, which refers to the quality of alternatives. An individual evaluates and compares their current relationship to other alternatives. If the alternatives, such as other potential partners, are of low quality, the commitment is higher as the current relationship is seen as the best option (48). The third factor that influences commitment is the investment, which is not a factor that has previously been considered in the social exchange theory (46, 48).

Investments are resources that have been put into a relationship, they can be intrinsic or extrinsic (7). Intrinsic investments are resources that directly contribute to the relationship and are usually non-materialistic such as time invested into the relationship or emotional work, but can also be materialistic such as money that is spent on the partner e.g., through gifts (7). Extrinsic investments are resources that are connected to the relationship and that exist because of it, and would be lost if the relationship ends. However, they are external and happen outside of the relationship (7). Examples of extrinsic investments are shared social circles, a social identity that emerged from the relationship, or shared material possessions. If the investment into a relationship is high, an individual stays in a relationship even if they are not satisfied by it. The combination of high satisfaction, few qualitative alternatives, and a high investment lead to a strong commitment to a relationship (48).

The investment model by (48) gives a base for understanding why people stay in abusive relationships. With a high investment and few alternatives, the low satisfaction of the relationship is disregarded (49).

### 3.2 Grooming strategies

Different studies have shown that groomers employ thought-through strategies to manipulate children into sexual relationships (50, 51). O’Connell (52) formulates the stages of the grooming process. These stages can be recognized in similar forms in various research on grooming stages and outline common behaviors of groomers. While these stages do not always occur in this fixed order, they can typically be observed in the following sequential pattern:

As a first step, the predator tries to form a friendship with the victim through exchanging general information and getting to know each other. After that, the groomer attempts to build a deeper relationship with the child. This is done by asking more personal questions about for example interests and hobbies. Risk assessment is the third step that can be seen in the typical grooming process. Here, the predator is asking questions about the minor’s environment to assess whether it is monitored by parents or other adults. When the risk is low that the child’s online activities are detected by its guardians, the offender starts to try to gain the victim’s trust. In this exclusivity stage, the conversations become more intimate and emotional, and a dependency is built between the predatory and the victim. Next, after that deeper connection emerges, the groomer introduces sexual topics to the conversation while engaging the child in it. As a last step, the offender attempts to meet the victim offline or to engage in online sexual activities with the minor (52).

Throughout the whole grooming process, based on the social exchange theory, the victim will only continue to communicate with the offender if the rewards of the relationship outweigh the costs (46). The perceived rewards for the victim can be emotional factors, such as attention, validation, support, and trust. In grooming cases, predators often make the victim feel like they are unique and special. Perceived costs of the child, in contrast, are the feelings of shame, guilt, stress, possibly anxiety, and in the case of a meeting offline physical harm.

The investment model emphasizes that the rewards and costs are not the only factors that influence whether one stays in a relationship (48). High investments make it harder for individuals to leave relationships even if they are abusive. Investments that are relevant for victims of online grooming are the time invested through chatting, or that they have shared deep personal secrets during the exclusivity stage where deep trust is built. This feeling of trust and exclusivity often makes the victim disregard other relationships with, for example, friends and family and they become isolated. As a consequence, this investment would lead to a loss of an important social contact that cannot be readily replaced. Moreover, because of the attention and compliments that the groomer gives to the victim, the victim’s self-worth and identity might be closely tied to their relationship.

All these investments can lead to a child not leaving the relationship even when the rewards no longer outweigh the costs. A victim can perceive that the relationship is no longer good and still stay committed.

### 3.3 Performance of existing approaches

Existing research evaluates the performance of different implementations using metrics such as accuracy, precision, recall, and the $F_{\beta }$ score (11). **Accuracy** is a metric used in classification problems to measure how many predictions are correct in proportion to total predictions. It shows how often the model predicts correctly (53).

$$\mathrm{Accuracy}=\frac{\mathrm{Number}\;\mathrm{of}\;\mathrm{Correct}\;\mathrm{Predictions}}{\mathrm{Total}\;\mathrm{Number}\;\mathrm{of}\;\mathrm{Predictions}}=\frac{TP+TN}{TP+TN+FP+FN}$$

**Precision** measures how many of the positive predictions are made correctly. It is the ratio of true positive predictions and all positive predictions (true positive predictions and falsely made positive predictions) (53). It gives an understanding of how many of the predictions identified as positive are correct. This measurement is especially important when it comes to grooming detection, as it shows how many detected grooming cases actually show predatory behavior. A low precision would mean that many non-grooming cases are labeled as predatory behavior which could lead to the unjustified damage of an innocent person’s image.

$$\mathrm{Precision}=\frac{\mathrm{True}\;\mathrm{Positives}}{\mathrm{True}\;\mathrm{Positives}\;+\;\mathrm{False}\;\mathrm{Positives}}$$

**Recall** is another metric that is used to measure a model’s performance. It is the ratio of correctly predicted positive instances to the amount of actual positive instances. A high recall would indicate that the model is good at detecting most positive instances (53). A high recall is important in grooming detection since missing predatory behavior is costly and can lead to child sexual abuse. Therefore, the recall needs to be considered in this research when evaluating a classification model’s performance.

$$\mathrm{Recall}=\frac{\mathrm{True}\;\mathrm{Positives}}{\mathrm{True}\;\mathrm{Positives}\;+\;\mathrm{False}\;\mathrm{Negatives}}$$

**$F_{\beta }$** is a score that describes the balance between the precision and recall metrics. A β value of 1 assigns equal weight to both precision and recall. A β below 1 puts more emphasis on precision compared to recall. The $F_{\beta }$ score measures the performance of classification models and is especially needed in cases when the dataset is unbalanced (53).

$$F_{\beta }=(1+\beta^{2})*\frac{\mathrm{Precision}*\mathrm{Recall}}{(\beta^{2}*\mathrm{Precision})+\mathrm{Recall}}$$

Research on grooming detection is based on chat log datasets that show a high imbalance between the predatory class and the non-predatory class. Grooming conversations are rare compared to conversations that show no predatory behavior. Research on grooming detection has put more emphasis on the precision than on the recall, that is why the $F_{1}$ and $F_{0.5}$ scores are the established metrics that are used to compare a model’s performance with other models. Accuracy is also looked at by other researchers when it comes to grooming detection performance but it holds no weight in this specific context as the dataset is highly imbalanced (11). Accuracy can be misleading in the case of an imbalanced dataset like PAN12, where only 3% of conversations have a predator as one of the authors. High accuracy can be achieved when the model focuses on the dominant (non-grooming) class and disregards the minority (grooming) class.

For grooming detection, there needs to be a balance between precision and recall to reduce the harm to both victims and innocent authors who are falsely detected as predators. False accusations are reduced by a high precision while a high recall prioritizes identifying predators. The $F_{\beta }$-score, describes this balance between precision and recall (54). As it is an ethical question of whether precision or recall holds more importance in the context of grooming detection, β=1 is a neutral option that puts equal weight on precision and recall. Therefore, the $F_{1}$ score is therefore the main indicator for the comparison between the performance of the different models in this research. Precision, recall, and $F_{1}$ also highlight what the models struggle with and indicate patterns in the data.

Tables 1, 2 present the current top-performing approaches for grooming detection. The PAN12 dataset is used in all of those papers. Table 1 describes which feature extraction technique and which classifier was used in the corresponding research. Table 2 displays the performances of the individual models. The grooming detection approach, as presented by Borj et al. (23) is the best performing one, based on the $F_{1}$-score. Fauzi et al. (13) and Nguyen et al. (14) both have an $F_{1}$-score of 0.98 and an $F_{0.5}$-score of 0.98, achieving excellent results.

### 3.4 Research setup

**Figure 1:**
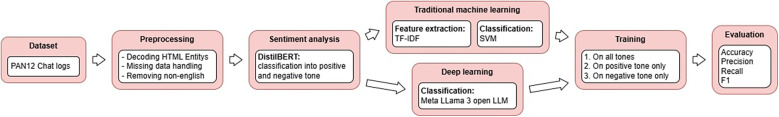
Analysis pipeline showing the steps taken from dataset to evaluation.

The process of gaining valuable insights into grooming detection and the strategies that are employed by groomers includes multiple steps (see Figure 1). The PAN12 dataset firstly needs to be preprocessed to remove noise and to extract important key features of the dataset. One of the objectives of this research was to examine how the tone of voice in grooming conversations affects the ability of machine learning models to detect predatory conversations. To achieve this goal, a sentiment analysis must be carried out. Conversations are sorted into sentiments on whether the tone of voice is positive or negative by the BERT language model. Multiple models were implemented in order to compare traditional machine learning with deep learning. As described in Section 2.5, SVM was chosen as the traditional machine learning implementation. To prepare the dataset for the training process, feature extraction was undertaken. The choice of the feature extraction method can highly influence the performance of grooming detection, as the SVM relies particularly on the input features to learn patterns (30). Through feature extraction, the raw data, which in this research are the chat messages, are converted into numerical values that are then compatible with the SVM. This is achieved through TF-IDF (Term Frequency – Inverse Document Frequency) vectorization. Research by Fauzi et al. (13) that compares multiple feature extraction methods in the context of grooming detection through SVM shows that TF-IDF captures the relevant data information best and achieves the best results in performance. TF-IDF vectorization is computationally efficient and was therefore chosen as the method used for feature extraction.

The deep learning model Llama 3, like other transformer-based large language models, in comparison does not need feature extraction, instead, it maps the input text directly to predictions. Tokenization was applied to split the raw text into smaller units (tokens) in preparation for the training process. The implementation of the traditional SVM and the LLama 3 large language model provided a baseline for comparing how different models perform in grooming detection. The classification task is the detection of conversations that contain grooming behavior. Both models were first trained on all tones of voice to see their overall performance. To later compare the impact of the tone of voice on the detection model, the models were trained on a conversation set that included only positive-toned grooming conversations and non-grooming conversations. This was then repeated with the negative-toned grooming conversations. As a last step, the performances of the different models were evaluated by comparing accuracy, precision, recall, and $F_{1}$ scores. In addition to these models, another LLaMA 3.2 1B model was fine-tuned to compare its performance to existing published models. The different models that are trained or fine-tuned in this research, their tasks and purpose are listed in Table 3.

**Table 3:** Description of models that are trained and fine-tuned.

Model	Train/test split	Training on	Purpose
SVM	Adjusted	All data	Comparison to LLM
SVM	Adjusted	Sentiments	Influence of sentiments
LLaMA 3.2	Adjusted	All data	Comparison to traditional ML
LLaMA 3.2	Adjusted	Sentiments	Influence of sentiments
LLaMA 3.2	Original	All data	Comparison to existing research

## 4 Results

This section describes the findings of the research. Firstly, the different steps of the research are described in more detail, including how the data was prepared for analysis. Subsequently, the different models’ configurations are listed and performance results are visualized. The Python programming language was used to preprocess the data, implement the classification models, and to evaluate them. The Sklearn library was used to implement the SVM classification models. The PyTorch framework was adopted for fine-tuning the LLaMA 3 model. For the implementation of the classification models, the National Academic Infrastructure for Supercomputing in Sweden (NAISS) was employed. The preprocessing of data and the training of the traditional machine learning model, the SVM, were possible on CPUs. However, GPUs were utilized to implement the LLaMA 3 LLM.

### 4.1 Preprocessing and labeling

The PAN12 dataset was preprocessed before the training of the various models to create a consistent dataset. Effective data preprocessing enhances the classification model’s performance and improves its robustness. Prior to preprocessing, the PAN12 dataset consisted of two separate datasets: a training dataset and a testing dataset. The training dataset consisted of 66,927 conversations with a total of 903,607 messages and 97,689 unique authors. The testing dataset consisted of 155,128 conversations with a total of 2,058,781 messages and 218,702 unique authors (see Table 4).

**Table 4:** Dataset before preprocessing.

Number of	Training dataset	Testing dataset	Total dataset
Conversations	66,927	155,128	222,055
Predatory conversations	2,016	3,737	5,753
Messages	903,607	2,058,781	2,962,388
Authors	97,689	218,702	307,693
Predators	142	254	394

The PAN12 dataset was created for a grooming detection competition and therefore the testing set is significantly larger than the training dataset. This allows for a more detailed comparison between competing models’ performances and subtle performance differences can be captured. For the LLaMA 3.2 model that serves as a comparison to previous models, this original split of training and testing data is kept to fine-tune the model, and to imitate the research setup of the existing contributions.

The dataset provided by PAN is formatted as an XML file. To facilitate easier processing, manipulation, and analysis of the data, it was first parsed into a pandas DataFrame. The preprocessing of data in this research was kept to a minimum to preserve linguistic information as much as possible. Over-preprocessing such as expanding abbreviations, stemming, or the removal of stop words, risked losing important elements that are unique to online chats and grooming conversations. Such features, abbreviations, and informal language are critical to understanding the patterns and tone in grooming conversations.

In XML files, characters like &, ’ or < are reserved characters that are described through HTML entities (e.g., & is represented through &). The first step of data preprocessing, therefore, was to decode these HTML entities back to their original characters. This ensured a clean, consistent and human-readable text for later training and analysis that aligned with natural language understanding. URLs, HTML tags, and extra spaces were removed as they do not hold meaningful linguistic value. Empty values were replaced with a space as a neutral placeholder. In this way the element could be kept while making it compatible with later tokenization steps. Finally, non-English conversations were removed, which aligns with previously done research. This was undertaken through the langdetect library that is based on Google’s language detection library (55). After preprocessing, the dataset consisted of 160,773 conversations with a total of 2,658,349 messages and 203,534 unique authors.

The PAN12 dataset includes files with IDs of conversations and corresponding messages that are considered to show grooming behavior. Moreover, all IDs of authors who are considered groomers are listed. This research is heavily focused on detecting patterns in groomers’ behaviors. Therefore the labeling process focused not only on the messages that were showing clear grooming but instead on whole conversations where a groomer was present. This approach shifts the attention to how groomers approach their victims. Early-stage interactions between groomers and their victims are relevant and provide valuable insights. Hence, all such conversations with a groomer present were labeled as predatory behavior and potential grooming. After the preprocessing and the labeling process, the dataset had 5,753 predatory conversations and 394 authors who were labeled as groomers (see Table 5).

**Table 5:** Dataset after preprocessing.

Number of	Total dataset
Conversations	160,773
Predatory conversations	5,753
Messages	2,658,349
Authors	203,534
Predators	394

The original goal of the PAN12 competition was the detection of groomers. It was less focused on deciding whether a conversation could be deemed to be of a grooming nature or not. Therefore, the authors were labeled based on the author IDs of the groomers only for the fine-tuning process of the LLaMA 3.2 model that was used as a comparison to earlier models.

### 4.2 Sentiment analysis

As the foundation for examining how tone sentiments affect grooming detection models, a sentiment analysis was performed using the DistilBERT model. To prepare the PAN12 conversations for the sentiment analysis the dataset was split into conversations that had a groomer present and those that did not.

Grooming conversations consist of a predator and a victim. Since these victims are adults pretending to be minors to lure these predators, this research does not focus on the messages that are sent by these adults imitating children. There are differences between the messages that adults pretending to be children send, and the messages that real children send (56). Only using messages for the sentiment analysis that are sent by predators, therefore, reduces potential deviations that can be caused by the “fake” children. While this could exclude potentially valuable information in the tone of the victim and how predators react to the behavior of victims, the decision was made to solely focus on groomer messages to maintain the authenticity of the data. All messages written by a groomer in a conversation were organized in a sequence and then tokenized. The DistilBERT model then predicted which tone of voice, positive or negative, was used. The conversation was then labeled positive or negative based on the result.

When it came to non-grooming conversations, all messages of a conversation were used as input for the sentiment analysis to capture the conversation’s full dynamic and exchange. Messages were organized as sequences, tokenized, and the label was predicted by the DistilBERT model. While this method introduces asymmetry, comparing groomers to full conversations, it was necessary to ensure the sentiment analysis is only done on authentic sources. Labeling non-grooming conversations by tone sentiment provides an understanding of how the tone of groomers compares and is different from the tone used by non-predatory users.

The results of the sentiment analysis can be seen in Table 6. The language that was used by groomers was positive in 46.85% of the conversations and negative in 53.15% of the conversations based on the DistilBERT analysis. When it comes to non-grooming, the tone was positive in 25.86% and negative in 75.14% of the conversations.

**Table 6:** Tone sentiments detected in conversations through BERT.

Sentiments	Positive (in%)	Negative (in%)
Non-grooming	40,081 (25.86%)	114,939 (74.14%)
Grooming	2,695 (46.85%)	3,058 (53.15%)
Total	42,776 (26.61%)	117,997 (73.39%)

Extracts of example conversations that are sorted into positive sentiment by the DistilBERT classifier can be found in Supplementary Appendix 1. Positive toned chats, while messages that are part of the negative sentiment can be seen in Supplementary Appendix 2. Negative toned chats.

### 4.3 Configurations

To compare the performance results of different kernels, an SVM was first trained with a linear kernel, and another one with an rbf (radial basis function) kernel, which is more complex and captures non-linear decision boundaries. To be able to fine-tune a LLaMA model, multiple training arguments have to be set. These hyperparameters include batch sizes, learning rate, number of training epochs, weight decay, and gradient accumulation steps. After testing multiple different configurations, the settings shown in Table 7 for the hyperparameters were used as they achieved the best performance.

**Table 7:** Hyperparameters of the LLaMA models.

Hyperparameter	LLaMA 3.2	LLaMA comparison model
Number of epochs	10	5
Batch size	32	32
Gradient accumulation steps	8	8
Learning rate	$1\times 10^{-5}$	$5\times 10^{-6}$
Weight decay	0.01	0.01

For both models, the SVM and the LLaMA, the PAN12 dataset was split into training and test sets, with 70% of the data belonging to the training dataset and 30% belonging to the validation dataset. These models were used to compare the performance of the SVM and LLaMA on the different sentiments. A 70/30 training-testing split provides a good balance between sufficient training data and adequate testing data. Lastly, to compare the newly released LLaMA 3.2 1B model with previous versions of the Meta model and previous research, the experimental setup of existing research was imitated for fine-tuning and validation of a third model that serves as a comparison to previous research. The PAN12 original split for training and testing dataset was kept and therefore makes the model’s performance metrics comparable to other grooming detection research.

### 4.4 Performance

The models were trained and evaluated on the PAN12 dataset as described before. Two different SVMs were trained, one with a linear kernel, and one with the rbf kernel. They were both initially trained on the whole training dataset, then they were trained on different grooming sentiments, in combination with non-grooming. Finally, they were tested on the different grooming sentiments.

The LLaMA 3.2 model with one billion parameters was also calibrated and evaluated on the PAN12 dataset. First, the model was fine-tuned to the whole dataset to compare its performance to the traditional SVM. Then it was fine-tuned and evaluated on the positive-toned grooming sentiment and non-grooming conversations. This process was repeated with the negative grooming sentiment. The optimal number of epochs was determined by minimizing the loss function, therefore the validation loss was examined and visualized. The LLaMA 3.2 model was fine-tuned one last time on the task of predator detection as a comparison model to existing research. In the following subsections, the performances of these different models are visualized.

### 4.5 Support vector machine

Two SVMs were trained on the PAN12 dataset, one with a linear kernel and one with a more complex rbf kernel. As previously described, the task was to detect predator conversations. The performance results of the SVMs trained and tested on the different sentiments are presented in Tables 8, 9. The training of the SVM models took approximately 15 min each.

**Table 8:** Performance of SVM with linear kernel on different sentiments.

Training	Testing	Accuracy	Precision	Recall	$F_{1}$	$F_{0.5}$	$F_{2}$
Positive + Negative	Positive + Negative	1.00	0.90	0.97	0.94	0.91	0.96
Positive	Positive	1.00	0.95	0.90	0.92	0.94	0.91
Negative	Negative	1.00	0.88	0.92	0.90	0.89	0.91
Positive	Negative	0.99	0.97	0.89	0.93	0.95	0.90
Negative	Positive	0.99	0.95	0.93	0.94	0.95	0.93

**Table 9:** Performance of SVM with rbf-kernel on different sentiments.

Training	Testing	Accuracy	Precision	Recall	$F_{1}$	$F_{0.5}$	$F_{2}$
Positive+Negative	Positive+Negative	1.00	0.98	0.94	0.96	0.97	0.95
Positive	Positive	1.00	0.99	0.85	0.91	0.96	0.87
Negative	Negative	1.00	0.99	0.87	0.92	0.96	0.89
Positive	Negative	0.99	0.99	0.82	0.90	0.95	0.85
Negative	Positive	0.99	0.99	0.85	0.92	0.96	0.87

Based on the performance results, the linear kernel is more suitable for the PAN12 dataset. While the precision is high when using the rbf kernel, the recall and $F_{1}$ scores are low. Since the PAN12 dataset is highly imbalanced, this indicates that the model is overfitting to the majority non-predatory class and only detecting very few predatory conversations.

### 4.6 LLaMA 3.2

**Figure 2:**
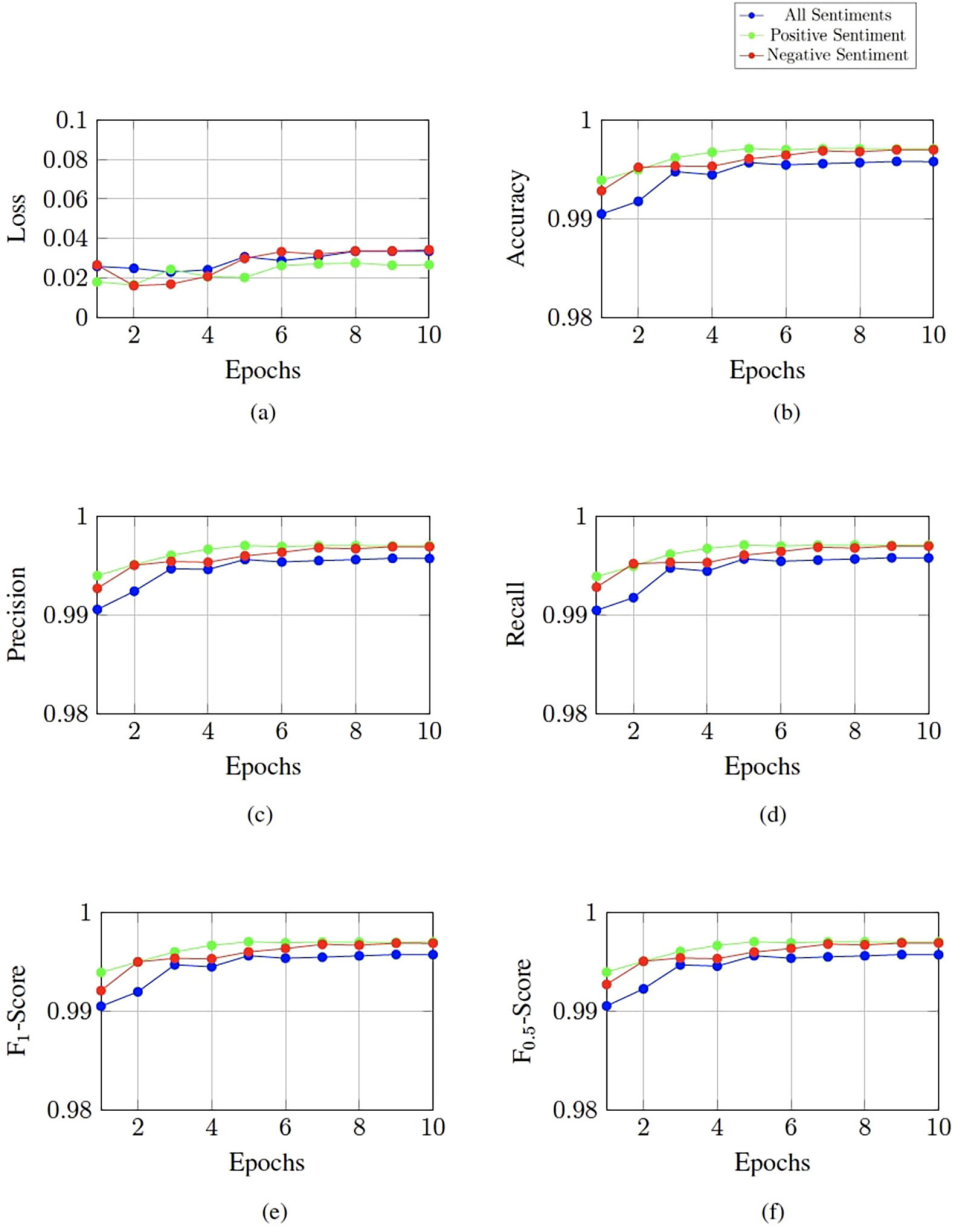
Performance of the LLaMA 3.2 1B model on different sentiments. **(a)** Validation loss over the epochs. **(b)** Accuracy over the epochs. **(c)** Precision over the epochs. **(d)** Recall over the epochs. **(e)** F_1_ score over the epochs. **(f)** F_0.5_ score over the epochs.

The LLaMA 3.2 1B model was fine-tuned to detect predatory chats with at least one predatory author. It was fine-tuned to the different tones of voices that have been detected through a sentiment analysis using the DistilBERT model. The LLaMA model was fine-tuned on all sentiments, the positive grooming sentiment together with non-grooming chats and the negative grooming sentiment together with non-grooming chats. The performance results of these three models after fine-tuning for 10 epochs can be found in Figure 2.

When fine-tuned on all sentiments, the model reached the lowest loss after three epochs. With an $F_{1}$ score of 0.99 and an $F_{0.5}$ score of 0.99. Fine-tuning on the positive sentiments led to a minimum loss after the second epoch with an $F_{1}$ score of 1.00 and an $F_{0.5}$ score. Similarly, the model that was fine-tuned on the negative sentiment reached the lowest loss after two epochs with the $F_{1}$ score being 1.00 and the $F_{0.5}$ score reaching 1.00 (see Table 10). Fine-tuning these LLaMA models took an average of about two hours each.

**Table 10:** Performance of LLaMA models when reaching the minimum of the loss function.

Sentiment	Loss	Accuracy	Precision	Recall	$F_{1}$	$F_{0.5}$	$F_{2}$
All sentiments	0.0229	0.99	0.99	0.99	0.99	0.99	0.99
Positive	0.0163	0.99	1.00	0.99	**1.00**	**1.00**	0.99
Negative	0.0160	1.00	1.00	1.00	**1.00**	**1.00**	**1.00**

Bold values indicate the highest values in a particular column.

### 4.7 LLaMA 3.2 as comparison model

A LLaMA 3.2 1B model was fine-tuned on the original split of training and testing sets of the PAN12 dataset. Its task was to detect predatory authors. The model reached the minimum of the loss function after two epochs of fine-tuning (see Table 11). Performance scores increased after the second epoch, however the model started overfitting to the training dataset after it had reached its lowest loss. After epoch two, the accuracy was 0.99, the precision 0.99, the recall 0.99, the $F_{1}$ 0.99, and the $F_{0.5}$ score 0.99. These performance scores are the basis for comparing the LLaMA 3.2 1B model with traditional machine learning models in existing literature and older versions of the LLaMA large language model.

**Table 11:** Performance of the LLaMA comparison model.

Epoch	Loss	Accuracy	Precision	Recall	$F_{1}$	$F_{0.5}$	$F_{2}$
1	0.0263	0.99	0.99	0.99	0.99	0.99	0.99
**2**	**0.0260**	**0.99**	**0.99**	**0.99**	**0.99**	**0.99**	**0.99**
3	0.0311	0.99	0.99	0.99	0.99	0.99	0.99
4	0.0344	0.99	0.99	0.99	0.99	0.99	0.99
5	0.0428	0.99	0.99	0.99	0.99	0.99	0.99

Bold values highlight when the model reached the minimum of the loss function.

The fine-tuning process of the LLaMA 3.2 1B model as a comparison model took approximately 4.5 h. Four A100 GPU cores were used.

## 5 Discussion

The aim of this section is to interpret the results of the implemented models. We discussed how the traditional machine learning model SVM compares to the large language model LLaMA for the detection of grooming conversations. Moreover, the influence of different tones within grooming approaches is discovered and described. The insights are explained through the application of the social exchange theory and the investment model. Lastly, the performance of the LLaMA 3.2 1B model in detecting predatory authors is compared to the performance of models found in existing literature.

### 5.1 Sentiments in grooming chats

As described in Section 2.7, the chat conversations of the PAN12 dataset are sorted into positive and negative sentiments based on the tone that is used by the authors through unsupervised sentiment analysis. While non-grooming conversations are labeled as positive sentiment in only 25.86% of the cases, grooming authors are using a positive tone in 46.85% of the conversations. In the PAN12 dataset, groomers, therefore, use a positive tone more frequently than authors in conversations that show no predatory behavior. While groomers may employ varying strategies, this typical behavior of groomers being overly positive can be explained through the social exchange theory. The social exchange theory states that an individual enters and stays in a relationship if the rewards of it outweigh the costs. Groomers establish trust with their victims to manipulate and sexually exploit them. As a result, groomers prioritize engaging their victims in conversation more than non-predatory authors do.

As the social exchange theory proposes, the child will enter and stay in the relationship if it perceives the costs to be less than the rewards. As mentioned, the costs of grooming for the child are large and include shame, guilt, anxiety and physical harm. Therefore, the groomer has to maximize the perceived rewards, which is achieved through giving the child attention, validation, trust, and the feeling of uniqueness. These affirmations are all enhanced through positive language, explaining why groomers use more positive language when compared to non-groomers.

In addition, the groomer uses positive language to increase the overall investment of the victim. Flattery and emotional closeness through positive language can lead to more engagement of the child and a higher time investment. Moreover, through excessive support, compliments and affection the child’s self-worth can be become more and more tied to the relationship with the groomer. Therefore, the self-worth of the victim can be seen as another investment that is enhanced through positive language. As shown in our sentiment analysis, groomers use abundant positive language to manipulate perceived rewards and investment, making the victim feel the benefits outweigh the costs, thereby keeping them in the relationship.

### 5.2 Comparing traditional ML and deep learning

The ability to detect grooming conversations is evaluated by comparing the performance scores of a traditional machine learning model (SVM) and a deep learning model (LLaMA 3) on the PAN12 dataset. The LLaMA model has a precision of 0.99 vs. 0.90 for the SVM. The higher precision of the LLaMA model demonstrates, that the conversations that are detected as grooming behavior actually show predatory behavior at a higher percentage than the conversations that are detected by the SVM. The LLaMA has a recall of 0.99 and the SVM of 0.97. The slight improvement of the recall with the LLaMA model also demonstrates that more grooming conversations are caught by the deep learning model than by the traditional SVM. The $F_{1}$ score of the LLaMA model equaling 0.99 means that precision and recall are balanced. While the model catches most predatory exchanges, the detected conversations are also truly predatory. The $F_{1}$ score of the SVM at 0.94 indicates a higher imbalance of precision and recall. The recall is high and a lot of grooming conversations are caught by the model. However, due to the precision-recall trade-off, the high recall of the SVM comes at the cost of misclassifying non-grooming conversations as grooming more frequently.

The LLaMA model can detect grooming conversations better than the SVM. However, this comes at the expense of needing high computational power, time and high memory requirements. The linear support vector machine can be trained on a single CPU core. The training and testing process of the SVM is completed within minutes. The LLaMA 3 model in comparison needs to be fine-tuned on multiple GPUs with large GPU memory (at least 40–80 GB) as the model, its weights, gradients, and optimizer states are loaded onto the memory. With four A100 GPU cores, fine-tuning the model to the PAN12 dataset takes circa two hours. When choosing between a traditional machine learning model or a modern large language model for grooming detection, one must decide whether the higher performance of the LLM is worthwhile.

### 5.3 Influence of tone

In this section we explore the effect of training both traditional machine learning models and deep learning models with positive and negative tone.

#### 5.3.1 SVM model

The linear SVM was first trained on all sentiments, it was then specialized onto the positive sentiment and then the negative sentiment of grooming conversations. The baseline model that was trained and tested on all sentiments reached a precision of 0.90, a recall of 0.97 and an $F_{1}$ score of 0.94 (see Table 8). When the model was trained and tested only on grooming conversations that were sorted into the positive sentiment and non-grooming conversations, the precision was 0.95, the recall 0.90 and the $F_{1}$ score 0.92. For the SVM that was trained and tested on the negative grooming sentiment and non-grooming conversations, the precision was 0.88, the recall 0.92 and the $F_{1}$ score 0.90. As the dataset is imbalanced, the accuracy is high even in cases when predatory conversations are not caught. As the models that are trained on the positive and negative sentiment show significant differences, it can be said that the positive and negative sentiments must be dissimilar in a way.

When looking at the $F_{1}$ score, which is used as the main metric to evaluate the models’ general performances, the SVM that was trained on all sentiments shows the highest performance. This indicates that the baseline model which was trained on both sentiments can capture patterns in grooming conversations that cannot be caught when the SVM was only trained on one sentiment. Patterns that are common between both sentiments are best detected when training on both sentiments.

Moreover, the $F_{1}$ score of the model trained with the positive sentiment is higher than the $F_{1}$ score of the model trained on the negative sentiment. A higher $F_{1}$ score shows a higher general performance of the SVM trained and tested on positive toned grooming conversations. Conversations with groomers who employ a more positive language are more easily differentiated from non-predatory conversations than grooming conversations that show a more negative tone. It seems to be harder for the SVM to distinguish between grooming conversations of the negative sentiment and non-grooming conversations, as its performance in terms of $F_{1}$ score is lower.

Furthermore, the precision is higher than the recall in the SVM trained and tested on the positive sentiment. In comparison, the SVM trained and tested on the negative grooming sentiment has a precision of 0.88 and a recall of 0.92. Unlike the positive trained and tested model, the recall is higher than the precision. When comparing the two models, the precision of the “positive” model is higher than the one of the “negative” model and the recall of the “negative” model is higher than the one of the “positive” model. The model trained and tested on the positive grooming sentiment therefore is more conservative in its predictions, meaning it is sure when labeling conversations as predatory behavior (as it can be seen with the high precision). However, it then misses some grooming cases leading to a lower recall score. The opposite is the case for the SVM that is trained and tested on the negative grooming sentiment. Here the model is more strict. It tries to catch a wider range of grooming conversations which is explained by the high recall. But therefore, more cases are detected as grooming behavior and conversations that are detected as such are not always truly grooming chats (see the lower precision score).

An explanation as to why conversations with groomers that use more positive language are more easily distinguished from non-grooming by the SVM can be found when applying the social exchange theory. As previously explained, predators use flattery, compliments, and affirmations to increase the perceived benefits of the relationship. When groomers follow this strategy and these tactics through overly positive language, the conversation fits the typical grooming approach and is therefore more easily detected.

#### 5.3.2 LLaMA 3 model

The fine-tuned LLaMA 3.2 1B models also show variations when trained on the different sentiments. All models achieve a low loss immediately after the first epoch, which shows that the pre-trained LLaMA model already has a good understanding of the language patterns in the chat logs. However, the point at which the loss function reaches its minimum during fine-tuning varies depending on the sentiment the model is trained for. The model that is fine-tuned on all sentiments reaches its minimum after three epochs of fine-tuning. The two models that are specifically fine-tuned on the separate sentiments both reach their lowest loss after two epochs. The model fine-tuned on the negative grooming sentiment and non-grooming chats has a loss that is slightly less but nearly identical to the loss of the “positive” model. The dataset that is used to fine-tuned the model on all sentiments is larger and has a wider variety of grooming chats, so that the model learns the patterns slower.

The model that was fine-tuned on all sentiments has an $F_{1}$ score of 0.99 (see Table 10). While these scores already are excellent and show a great ability to detect grooming conversations, the models that are fine-tuned on the separate sentiments score a higher $F_{1}$. Both the positive fine-tuned and the negative fine-tuned models achieve $F_{1}$ scores of 1.00. These results show a slight improvement of performance compared to the model fine-tuned on all sentiments.

These higher F-scores suggest that there is a structural difference between the conversations in the positive and the negative sentiment. Distinct linguistic patterns in these two sentiments make it harder for the model that is fine-tuned on both sentiments to generalize across the sentiments, resulting in a worse performance. This confirms the assumption that was made when analyzing the results of the SVMs, that patterns in positive and negative toned chats are dissimilar from each other.

Furthermore, the recall of the model fine-tuned on the positive sentiment was slightly less than the recall of the LLaMA model that was fine-tuned on the negative sentiment. The same observation was made when looking at the precision and recall of the SVMs that are trained on the separate sentiments. Although the differences in scores of the LLaMA models are only slight, they confirm that the model fine-tuned on the positive sentiment is more conservative in its predictions, while the one fine-tuned on the negative sentiment is stricter. This is also the case for SVMs, which indicates that grooming approaches that use positive language are more distinct from non-grooming than grooming approaches that use overall negative language.

While the SVM that is trained on all sentiments can better catch all complex patterns of grooming chats than when trained on separate sentiments, the LLaMA 3.2 model can better understand the nuanced patterns of positive and negative toned grooming conversations when fine-tuned to the distinct sentiments. This is likely due to the less complex structure of the SVM. Moreover, the results of the different models suggest that conversations where groomers make use of positive language are more easily distinguishable from non-predatory conversations for machine learning models. Grooming conversations where an overall negative language is used, however, are more nuanced and have more complex patterns that are harder to tell apart from non-grooming chats.

Based on the social exchange theory groomers commonly use positive language to increase the perceived rewards through compliments and support. As seen in the results of the models, when this approach is used by groomers, the predatory conversations are more easily caught by machine learning models. However, the finding that positive tone grooming differs from negative tone grooming suggests that groomers using negative language employ a distinct strategy. Threats, criticism, blame, gaslighting and insults are examples of negative language. When using this kind of language, the predator is trying to increase the perceived investments as described in the investment model. When the investments are high enough in a relationship, a partner will not leave the relationship even if the satisfaction is low. Groomers that are using negative language could aim to make their victims feel guilty and responsible for the relationship so that the victim feels obligated to stay as they feel that they have already invested into the relationship.

These two different strategies could explain how the negative toned and positive toned grooming approaches differ from each other. Previous research on grooming approaches has focused on creating grooming stages that should fit onto all grooming cases. In existing research, the emphasis is based on the assumption that groomers primarily aim to gain the trust of the victim. However, as shown in this study, not all groomers’ strategies are to build trust through being overly positive and instead grooming approaches show variations. Patterns that are used when groomers use negative language are not the same as the patterns that are used when groomers use a more positive language. Therefore, the results of this research suggest that there is not one grooming strategy that is used by all groomers but that there are multiple strategies.

### 5.4 Comparison of LLama 3.2 with models in existing research

The LLaMA 3.2 1B model outperforms both traditional machine learning, as well as the older version of the LLaMA large language model. LLMs like the LLaMA 3.2 are able to understand more complex language patterns than traditional machine learning models and are more suited for the task of grooming detection where manipulation is a big component. In Table 12, the LLaMA 3.2 1B’s performance metrics are compared to the best-performing approaches found in the literature. Moreover, the LLaMA 3.2 1B is best suited for detecting grooming, as it not only delivers the highest performance but is also easy to implement, requiring minimal preprocessing and no feature extraction. Finally, it is smaller and requires less computational resources than other large language models like the LLaMA 2.

**Table 12:** LLaMA 3.2 1B performance compared to existing approaches.

Reference	Accuracy	Precision	Recall	$F_{1}$	$F_{0.5}$	$F_{2}$
Borj et al. (23)	0.99	–	–	**0.99**	0.94	–
Fauzi et al. (13)	0.98	0.98	0.98	0.98	0.98	0.98
Nguyen et al. (14)	1.00	–	–	0.98	0.98	–
LLaMA 3.2 1B	0.99	0.99	0.99	**0.99**	**0.99**	**0.99**

Bold values indicate the highest values in a particular column.

## 6 Conclusion

Grooming is a ubiquitous and concerning issue, it is important to detect predatory behavior to protect children in online spaces. Many approaches have been implemented to automatically detect grooming behavior in chat logs, that range from traditional machine learning to large language models. Large language models have revolutionized natural language processing with their ability to understand complex language patterns.

We have compared traditional machine learning models with new deep learning models. To accomplish this, the traditional support vector machine was compared to the LLaMA 3.2 large language model by Meta. Both models were trained or fine-tuned to detect grooming conversations in the PAN12 dataset. While both models performed well, the $F_{1}$ score indicates that LLaMA is more effective at detecting grooming chats than SVM. This suggests that deep learning better captures the complex patterns of grooming compared to traditional machine learning. Deep learning models, such as the implemented LLaMA model, however, require more computational power and memory than traditional machine learning such as SVMs.

Furthermore, we explored whether the tone used by predators impacts the effectiveness of grooming detection. The grooming chats were first sorted into sentiments based on the DistilBERT model. The resulting sentiments show that groomers often use a more positive language when communicating with their victims, confirming Bogdanova et al. (57). Subsequently, SVMs and LLaMA 3.2 models were implemented which were first trained/fine-tuned on all sentiments, then on the positive grooming sentiment, and lastly on the negative sentiment. While the SVM was not able to detect grooming as well when trained on specific sentiments, than when trained on all sentiments, the LLaMA’s performance improved when fine-tuning on the separate sentiments. We found that chats with positive sentiment exhibited varying patterns than those with negative sentiment. The precision and recall scores of both the SVM and the LLaMA indicate that chats where groomers use a positive language are more easily distinguishable from non-predatory chats for the models. This leads us to believe that negative toned grooming chats are more nuanced and complex. The performance differences between tones observed in this research should be further tested in future research on other datasets to assess the generalizability and significance of the results.

In this research, we analyzed the overall tone of entire conversations. Future work could further explore how tone evolves throughout the course of the conversation, which may reveal more insights into grooming behavior. Additionally, the sentiments used in this research are positive and negative. A more nuanced distinction of sentiment and emotional markers could give more clues about the tone influences classification models (57).

Lastly, to compare the newly released LLaMA 3.2 1B large language model with models used in existing research on grooming detection, the original setup of the PAN12 competition was imitated which allowed for a fair comparison. The LLaMA 3.2 1B model outperformed both traditional machine learning models and its predecessor, LLaMA 2 7B, in detecting grooming. Large language models are constantly improving and providing better and more accurate ways to process complex language such as grooming chat logs.

Our findings contribute to a better understanding of grooming behavior. When understanding the patterns and strategies that are used by predators, more grooming cases can be caught and children are better protected in online spaces. LLaMA 3.2’s strong performance offers an effective method for detecting grooming in chat logs, aiding in the identification of groomers.

## 7 Ethical considerations

Grooming and sexual abuse of minors are sensitive topics that need to be handled with caution. However, the PAN12 dataset allows ethical research on grooming. The dataset is widely used by researchers and openly available. The authors of the PAN12 dataset are anonyomized to protect their identities, which makes the dataset compatible with current EU regulations (2). Moreover, the chat logs consist of predators and adults who pretend to be minors, and no real children are involved in this research. Therefore, the comparison of different grooming detection models and the investigation of grooming strategies on the PAN12 dataset do not pose any ethical problems.

A real-life implementation of grooming detection models, however, needs further ethical considerations. False positives harm innocent users, while false negatives have tough consequences for victims that can lead to child abuse. Online grooming is illegal in most countries around the world and in some countries, the use and obtainment of real chat logs that show grooming behavior is forbidden (5). Care must be taken when considering what data can be used to train grooming detection models. Another issue is that predators tend to contact their victims through private messages (51). Monitoring, surveillance, and analysis of such private chats is a violation of privacy for users of these platforms.

The implementation of LLMs in real grooming cases, especially those provided by big companies such as Meta or Google, raises privacy concerns. While generally input data is not stored, Meta’s responsible use guideline for LLaMA states that input of private and sensitive data should be restricted (58). To reduce these ethical concerns around the possible logging of input data, fully offline environments should be preferred for fine-tuning models.

## Data Availability

Publicly available datasets were analyzed in this study. This data can be found here: https://zenodo.org/records/3713280.
