# Supplementary material for: Comparing machine learning models with a focus on tone in grooming chat logs
Source: Front Pediatr. 2025 Jun 19;13:1591828. doi: 10.3389/fped.2025.1591828 (PMC12222207; doi:10.3389/fped.2025.1591828)
Supplement: Supplementary file 1 [file Datasheet1.pdf]

1 POSITIVE TONED CHATS

**Predator:**	i think u like me
**Victim:**	ya ur rlly nice to me
**Predator:**	i like u is why
**Predator:**	dont u know how much i like u
**Predator:**	i always tell u
**Victim:**	:)
**Predator:**	what r u think
**Predator:**	thinking
**Victim:**	just gettin kinda slpy
**Predator:**	ok
**Predator:**	nite nite my sweet
**Victim:**	u goin to sleep?
**Predator:**	i thought u was
**Victim:**	soon
**Predator:**	ok
**Predator:**	think about me before u go to sleep
**Predator:**	i will u
**Victim:**	k
**Victim:**	aww
**Predator:**	u r my baby
**Predator:**	:-*
**Victim:**	thats so sweet
**Predator:**	i mean it
**Predator:**	u r special
**Victim:**	:)
**Predator:**	:D
**Victim:**	:D
**Predator:**	u still chatting to that guy
**Victim:**	na he buzzed me
**Victim:**	n i said hes rude
**Victim:**	n he stopped lol
**Predator:**	ok
**Predator:**	i am going back to bed
**Victim:**	aww k
**Predator:**	i will think about u until i fall asleep
**Victim:**	awwww
**Victim:**	nite
**Predator:**	nite my beautiful sweetheart

**Predator:**	hey sexy how was your day good i hope

**Predator:**	hello my sexy little lady how was your day hope to talk to u latter well hope your day was wonderful :D
