# Supplementary material for: Comparing machine learning models with a focus on tone in grooming chat logs
Source: Front Pediatr. 2025 Jun 19;13:1591828. doi: 10.3389/fped.2025.1591828 (PMC12222207; doi:10.3389/fped.2025.1591828)
Supplement: Supplementary file 2 [file Datasheet2.pdf]

1 NEGATIVE TONED CHATS

**Predator:**	so u dont want me come over on that weekend
**Victim:**	yes i do!
**Predator:**	ok i am confuse
**Predator:**	i am not planning on bee their until then
**Victim:**	so y is a problem if i just tell u next the week u will come?
**Predator:**	i just like 2 b plan so everthing is set 4 that time that is all
**Predator:**	it is just me
**Victim:**	bt that will still give u lots of time
**Predator:**	yes it would
**Predator:**	just want things 2 b right
**Victim:**	kk
**Predator:**	do u blame me 4 thinking that way
**Predator:**	i do understand i really do
**Predator:**	i just wondering if u trust me
**Victim:**	i need to go cauz my mom wants to go eat i will bb tonite im srry bye for now

**Predator:**	you must be mad at me now... hav a great night

**Predator:**	when are u ever gonna be online

**Predator:**	hi princess
**Predator:**	i guess u dont want to chat with me
**Predator:**	fine
**Predator:**	why wont u chat with me
**Predator:**	r u busy
**Predator:**	i know u r invisible
**Predator:**	fine be that way
**Predator:**	nite
**Predator:**	i am going to bed
**Victim:**	i wasnt here i wouldnt not answer u
